# Balancing Care and Sacrifice: Lived Experiences and Support Needs of Primary Caregivers in Pediatric Chronic Pain Across Canada and Australia

**DOI:** 10.3390/children12070911

**Published:** 2025-07-10

**Authors:** Nicole Pope, Nicole Drumm, Kathryn A. Birnie, Melanie Noel, Carolyn Berryman, Nicki Ferencz, Tieghan Killackey, Megan Macneil, Darrel Zientek, Victoria Surry, Jennifer N. Stinson

**Affiliations:** 1 Child Health Evaluative Services, Research Institute, The Hospital for Sick Children, Toronto, ON M5G 1E8, Canada; jennifer.stinson@sickkids.ca; 2 Department of Nursing, Melbourne School of Health Sciences, Faculty of Medicine, Dentistry, and Health Sciences, The University of Melbourne, Melbourne, VIC 3052, Australia; 3 Murdoch Children’s Research Institute, Melbourne, VIC 3052, Australia; 4 Lawrence S. Bloomberg Faculty of Nursing and IHPME, University of Toronto, Toronto, ON M5S 1A1, Canada; nicole.drumm@sickkids.ca (N.D.); tieghan.killackey@utoronto.ca (T.K.); 5 Department of Psychology and Neuroscience, Faculty of Science, Dalhousie University, Halifax, NS B3H 4R2, Canada; kathryn.birnie@ucalgary.ca; 6 Department of Anesthesiology, Perioperative, and Pain Medicine, University of Calgary, Calgary, AB T2N 1N4, Canada; 7 Department of Community Health Sciences, University of Calgary, Calgary, AB T2N 1N4, Canada; 8 Department of Psychology, University of Calgary, Calgary, AB T2N 1N4, Canada; melanie.noel@ucalgary.ca; 9 Alberta Children’s Hospital Research Institute, Hotchkiss Brain Institute, Calgary, AB T2N 4N1, Canada; 10 Pediatric Chronic Pain Service, Women’s and Children’s Hospital, Adelaide, SA 5006, Australia; carolyn.berryman@adelaide.edu.au (C.B.); nicki.ferencz@sa.gov.au (N.F.); 11 Innovation, Implementation and Clinical Translation (IIMPACT) in Health, UniSA Allied Health & Human Performance, University of South Australia, City East Campus, Adelaide, SA 5001, Australia; 12 Faculty of Health and Medical Sciences, School of Psychology, University of Adelaide, Adelaide, SA 5005, Australia; 13 Peter Munk Cardiac Centre, University Health Network, Toronto, ON M5G 2C4, Canada; 14 Chronic Pain Network, McMaster University, Hamilton, ON L8S 4L8, Canada; megan.macneil@ucalgary.ca; 15 School of Public Health, University of Alberta, Edmonton, AB T6G 2R3, Canada; 16 Independent Researcher, Ottawa, ON K2J 4W5, Canada; lcolzman@gmail.com; 17 Faculty of Human and Social Development, School of Public Administration, University of Victoria, Victoria, BC V8P 5C2, Canada; victoriasurry@uvic.ca

**Keywords:** pediatric chronic pain, caregiver well-being, mental health, family-centred care, digital health interventions, virtual support, qualitative research, co-design

## Abstract

Background: Chronic pain affects one in five youth globally and is frequently accompanied by mental health challenges that extend into adulthood. Caregivers play a vital role in supporting youth with chronic pain, yet their own mental and physical health needs are often overlooked. While caregiver well-being is linked to child outcomes, few interventions directly address caregivers’ health, especially among those facing systemic barriers. This study explored the lived experiences of caregivers to better understand their unmet needs and inform the co-design of a supportive digital health solution. Methods: We conducted a qualitative exploratory study involving 32 caregivers of youth with chronic pain across Canada and Australia. Semi-structured interviews were co-facilitated by caregiver partners. Thematic analysis was applied to interview data. Results: Two overarching themes were identified: (1) bearing the weight and sacrifice of caregiving and (2) deep interrelatedness and blurred boundaries. Caregivers reported profound emotional, physical, and financial burdens; strained relationships; and social isolation. Many struggled with self-neglect, prioritizing their child’s needs over their own. Fathers’ evolving caregiving roles challenged traditional gender norms, though mothers continued to bear a disproportionate load. Despite challenges, caregivers demonstrated resilience and recognized their well-being as interconnected with their child’s health. Conclusions: Findings underscore the need for systemic investment in caregiver well-being. Digital health solutions, including virtual peer networks, mental health resources, and tailored education, offer scalable, accessible pathways for support. These insights will inform the development of Power over Pain for Primary Caregivers, a digital solution and knowledge hub aimed at improving caregiver well-being and family outcomes, aligning with global efforts to enhance family-centred pediatric pain care.

## 1. Introduction

Chronic pain in youth is a significant and complex public health crisis affecting one in five children and youth globally [1], often co-occurring with life-threatening mental health issues (e.g., suicidality and opioid misuse) that can persist into adulthood [2,3], and far exceed the rates of their healthy peers. Caregivers are critical to optimal health outcomes for children with chronic pain, yet their health needs are neglected [4]. While pediatric pain care focuses on the child, caregivers, often parents, suffer high levels of chronic stress and poor physical and mental health themselves [4,5,6]. Many also face challenges in their family relationships and parenting [7]. Poor caregiver health is a strong predictor of adverse child outcomes and overall family functioning [7,8]. Although some caregiver-focused interventions exist, they primarily target parenting behaviors (e.g., reducing overprotectiveness) rather than directly addressing caregivers’ mental health, chronic stress, or physical well-being, including their own chronic pain [9]. This gap is critical, as parental health is one of the most powerful predictors of child pain outcomes and treatment responsiveness [8]. For example, high levels of parental stress hinder a child’s responsiveness to psychological treatment for pain [10].

Despite the urgent need for caregiver-targeted support, most caregivers never receive the resources they need [4,11]. This gap is further pronounced for marginalized families, including single-parent, immigrant, refugee, LGBTQ+, and racialized caregivers, who face systemic barriers such as stigma, financial hardship, and limited healthcare access [5,6,7,12]. To address access barriers, our team developed the Power over Pain Portal (*PoP*) [https://www.popyouth.ca/ (accessed on 10 May 2025)] [13], a digital health solution leveraging Health Canada’s Wellness Together program [14], to provide evidence-based virtual support for youth with chronic pain. Using the Stepped Care model [15], *PoP* integrates self-assessment tools, pain neuroscience education, web- and app-based cognitive behavioral therapy strategies, and virtual peer support. This scalable, accessible platform presents a significant advancement in addressing the needs of youth with chronic pain, integrating free, evidence-based pain management and mental health support.

While *PoP*’s formal evaluation is ongoing, its early implementation has demonstrated the feasibility of delivering stepped, evidence-based virtual care at scale. Our team identified a persistent gap in caregiver-specific virtual support tools [9,16]. Both caregivers and youth have emphasized that caregiver well-being is a top care priority [17,18], yet few interventions directly address caregivers’ own mental and physical health needs, including chronic pain [4,9]. Early insights from *PoP*, such as user feedback on accessibility, the integration of mental health resources, and calls for caregiver-specific content, have highlighted the need for a complementary digital solution focused on caregivers. Our recent scoping review further underscored that existing digital resources overwhelmingly target parenting techniques rather than practical supports, respite care, or the systemic inequities that disproportionately affect marginalized caregivers [9]. Evidence shows that strengthening caregiver well-being can lead to improved outcomes for children and families [4,8,19], reinforcing the need for innovative, equity-oriented caregiver supports in virtual care ecosystems.

Building on evidence and *PoP*’s success, this study explores the lived experiences and support needs of caregivers of children with chronic pain through qualitative interviews with diverse caregivers in Canada and Australia. The aim of this study was to explore these lived experiences, with a particular focus on caregivers’ mental and physical well-being. Unlike prior studies that focus primarily on parenting strategies or stress regulation, our study centres caregiver well-being as a critical health equity issue, with attention to the gendered, cultural, and systemic dimensions of care. It is the first to integrate cross-national perspectives with caregiver-partnered data collection to inform the co-design of Power over Pain for Primary Caregivers (*PoPPc*), a digital health solution and knowledge hub. This work adds new understanding of caregiver identity, self-care, and digital needs, aligning with national and global priorities to enhance equitable, family-centred pediatric pain care [18,20,21].

## 2. Methods

### 2.1. Participants

This study, grounded in the principles of naturalistic inquiry and a constructivist worldview [22], employed a qualitative exploratory design [23] to capture the perspectives of a diverse (varying sex, gender, racialized identity, sexual orientation, single vs. partnered parental role, and rurality) group of Canadian and Australian primary caregivers (hereafter “caregivers”) of children with chronic pain to understand their mental health and well-being needs. We used active strategies to purposively recruit caregivers from chronic pain clinics across Canada and Australia. The diverse referral sources at these chronic pain clinics supported the reach and engagement of various populations historically excluded from research (i.e., rural and remote areas, single-parent households, and caregivers of gender-diverse children). Community caregivers of children with chronic pain were reached through our partnership with Solutions for Kids in Pain (SKIP) and public study promotion via social media campaigns from the participating pain clinics. Caregivers were eligible if they (a) had a child aged 12–18 years with chronic pain and (b) spoke and read English. Caregivers were excluded if they self-reported untreated major psychiatric illnesses (e.g., schizophrenia) and/or active suicidality at the time of screening, precluding their ability to participate in a verbal interview.

### 2.2. Procedure

The study was conducted in accordance with the Declaration of Helsinki [24] and approved by the institutional review boards (approval IDs RCH: HREC#2024-28701-51373-5; WCH: 2024/HRE00105 and SickKids: REB # 1000081307). Reporting is per the Consolidated Criteria for Reporting Qualitative Research checklist [25].

Following research ethics approval in both Canada and Australia, separate electronic consent and demographic surveys were developed and distributed via email to eligible participants in each country, taking into account region-specific contexts and institutional requirements. This allowed for customizing survey content and administration processes aligned with the respective institutions’ ethics and privacy standards. Demographic data collected included age, sex, gender, racialized identity, type of chronic pain, sexual orientation, socioeconomic status, language, rurality, and family composition. To adhere to local data storage and governance policies, surveys were hosted on REDCap^®^ [26] with separate surveys managed by each institution (removed for peer review). This facilitated the collection of comparable data while maintaining adherence to ethical guidelines [27].

Interview guides containing semi-structured questions were developed in collaboration with an interdisciplinary team of academics, clinicians, and primary caregiver advisors (Supplementary Materials). Interview guides were pretested with a caregiver of a 12-year-old child living with chronic pain who did not participate in the study. This pretesting led to refinements for clarity and the removal of redundant questions. Interview questions focused on exploring caregivers’ experiences of caring for a child with chronic pain, their mental and physical health, well-being needs, how they navigate caregiving challenges, and their resource needs. The interview questions were iteratively refined throughout the study based on analysis of preliminary interviews to enhance clarity and comprehension of the questions and explore new concepts as they unfolded [28], such as the role of male caregivers and the influence of caregiving on the child–caregiver relationship.

Online interviews were conducted in English between June and December 2024 by the first author (NP), a registered nurse and postdoctoral researcher, who is a woman with extensive training and experience in qualitative research. Her background in pediatric nursing, commitment to improving pain care for children, and personal perspective as a mother motivated her to undertake this work. A research officer (AN), a psychology student skilled in qualitative research, conducted some of the interviews. Our primary caregiver partners (DZ, male; MM, female) co-hosted interviews with these researchers, ensuring that lived experiences deeply informed the study. These advisors, experienced in caregiving a child with chronic pain, brought a distinctive peer perspective to the interview process and fostered a sense of trust and relatability between participants and the research team, allowing participants to feel understood, validated, and supported during the interview [28]. This approach enriched the interview dynamics by offering peer-to-peer connections and creating a supportive environment for participants to share their experiences openly and authentically [29]. Caregiver partners were compensated according to best practices to acknowledge their expertise and contributions [30,31].

Interviews were conducted via secure video conferencing platforms (Zoom for Healthcare), ensuring participant confidentiality and enabling a flexible, accessible method of engagement [32]. This approach allowed participants to select a time and setting that best suited their caregiving responsibilities, thereby minimizing disruptions to their routines. In keeping with a semi-structured interview approach [33] and the exploratory study design [23], the interviewers used open-ended questions and prompts to encourage detailed responses (e.g., “Can you tell me more about that?”), paraphrased to ensure clarity (e.g., “Am I understanding this correctly?”), and adjusted the flow of conversation as needed to ensure participant comfort and natural dialogue. AN’s academic background in psychology complemented this approach, bringing an additional lens of understanding to the mental health and well-being aspects of each interview. The primary caregiver advisors’ lived experience added depth to the interaction, creating an empathetic and inclusive environment that resonated with participants’ perspectives.

Leveraging the online format and the involvement of caregiver co-hosts ensured interviews successfully captured nuanced insights into caregivers’ mental, physical health, and well-being needs. All interviews were audio-recorded with participant consent. To maximize data immersion [34], all audio-recorded interviews were transcribed verbatim and proofread by NP or AN alongside the original recordings to ensure accuracy. Interview transcripts were uploaded, organised, and managed in NVivo [35]. All data were securely stored and managed in accordance with institutional privacy policies. Interview transcripts were de-identified, and all potentially identifying details were removed during transcription and reporting to ensure participant anonymity.

### 2.3. Data Analysis

We employed qualitative thematic analysis [34] to systematically analyze interview data through an iterative process of inductive and deductive coding, categorization, and conceptualization, guided by the biopsychosocial model [36] and family-centred pain care theories [37]. The unit of analysis was the interview text, focusing on latent content (underlying meanings, patterns, and interpretations beyond what was explicitly stated) to identify patterns and themes [38]. Two authors (NP and ND) conducted multiple readings of the interview transcripts to familiarize themselves with the data, gain a holistic understanding of it, and develop preliminary coding notes [34]. Regular meetings were held to discuss patterns and refine the early stages of analysis reflexivity [39]. NP is a clinician-researcher with expertise in qualitative research and a special interest in supporting families and children with chronic pain. Drawing on the biopsychosocial model [36] and family-centred pain care theories [37], our deductive approach was informed by established knowledge about the impact of caregiving on families and children with chronic pain. Both authors inherently brought an emic perspective to the analysis, offering unique insights into the study [39]. The authors took deliberate steps to mitigate their pre-existing expectations to ensure that participants’ perceptions guided the findings. For example, the authors regularly discussed coding and developed themes with the broader research team, who were less involved in data collection, supporting the credibility of the analysis [40]. The integration of theoretical perspectives helped ensure that findings were contextualized within broader frameworks of caregiving and pediatric pain management. Our primary caregiver advisors, who have lived experience, also provided input on preliminary findings to ensure the themes resonated with real-world caregiver experiences.

Relevant passages from the transcripts were divided into meaning units, condensed, abstracted, and labeled as codes [34]. Interview data were reviewed individually and collectively to identify shared and differing patterns. NP and ND independently coded each transcript; coding was verified by a second coder for accuracy and to ensure contextual integrity [40]. Differences in interpretation, such as differing judgments about how participants described their health needs or caregiving challenges, were resolved through reflexive discussions with the broader study team [38,40]. Codes were aggregated into categories, subsequently reviewed, and refined into final, broader themes [34]. These themes were conceptualized deductively using theoretical insights from the biopsychosocial model and family-centred pain care theories and inductively based on the participants’ lived experiences. Reflexivity ensured that the authors remained close to the data while acknowledging the interpretive nature of qualitative inquiry [38,40]. These sessions offered opportunities for rigorous examination of the codes, categories, and final themes. We leveraged the team’s diverse expertise in clinical practice, pediatric pain management, and research to ensure that the findings accurately reflected participants’ experiences and were grounded in data. Representative participant quotations are included in each theme to illustrate the findings and enhance the trustworthiness of the results [40].

The principles of information power guided sample size requirements [29]. This approach evaluates five items: study aim, sample specificity, theoretical foundation, dialogue quality, and analysis strategy, to determine when sufficient data has been gathered [29]. The research team iteratively assessed these items throughout the research process and concluded that a sample of 32 participant interviews provided adequate information power to address the research questions. The richness of the data was enhanced by purposive sampling, a focused study aim, and the inclusion of caregivers with diverse caregiving experiences related to youth chronic pain. Additionally, our primary caregivers, who have lived experience, were actively engaged in the project, serving as co-hosts for many of the interviews. By applying the biopsychosocial model [36] and family-centred pain care theories [37] as our guiding frameworks, we were able to comprehensively examine the complex interplay between children’s chronic pain, family roles, and caregiving experiences. Dialogue quality was robust, evidenced by strong rapport, high participant engagement, and naturally flowing conversations focused on the study objectives [29]. Our theoretical approach ensured that both individual and systemic caregiving challenges were captured and interpreted within a well-established conceptual framework. Our collaborative and inclusive approach, grounded in biopsychosocial [36] and family-centred theories [37], resulted in data that were rich in depth and breadth, providing nuanced understandings of caregivers’ roles, challenges, and needs as caregivers for their children living with chronic pain.

## 3. Results

A total of 32 caregivers from across Canada and Australia were interviewed, with 17 interviews co-hosted by a primary caregiver partner advisor (DZ or MM). The mean age of primary caregivers was 48.6 years (range: 30–59), and the mean age of their children was 14.7 years (range: 11–21). Most participants (n = 21, 66%) identified as female. Participants represented diverse ethnic backgrounds, with the majority identifying as White or European, while others identified as Aboriginal/First Nations, Black, Chinese, South Asian, or mixed race. Table 1 summarizes the participant demographic characteristics. The mean interview duration was 44 min (range: 24–79 min). The research team organized qualitative results into two broad themes (Table 2) labeled: (1) bearing the weight and sacrifice of caregiving and (2) deep interrelatedness and blurred boundaries. Together, these themes reflect how caregiving for a child with chronic pain reshapes personal identity, family dynamics, and caregivers’ capacity for self-care within systems that offer limited support. These themes were actively constructed through reflexive, iterative analysis that integrated participant narratives with theoretical insights from the biopsychosocial model and family-centred care frameworks. These themes and corresponding subthemes are presented below with supporting participant narratives. All potential identifiers have been removed.

**Theme 1: Bearing the Weight and Sacrifice of Caregiving**

This theme encapsulates the profound emotional, social, and personal toll that caregivers experienced, as well as their resilience, adaptability, and dedication. It reflected caregivers’ stories about navigating paths marked by intense personal sacrifice, emotional strain, and isolation from social and professional circles. It captured caregivers’ sentiments about how they prioritized their child’s needs and underwent a redefinition of their identities and relationships. Spouses drifted apart, friendships often weakened and shifted, and attention to siblings was sometimes compromised. Amidst these challenges, caregivers found moments of deepened bonds and a sense of purpose. They balanced self-sacrifice to support their family and sought connection in shared experiences.

### 3.1. Redefined Relationships and Roles

Caregivers described how caregiving responsibilities were often distributed unequally within families, with one parent, typically the mother, taking on the majority of the daily caregiving tasks. These role divisions were shaped by work commitments, gender expectations, and logistical constraints, often leading to an imbalance in caregiving load. While fathers and other family members provided support, mothers frequently found themselves carrying the heavier burden of daily care and medical advocacy. One father shared how his wife inherently carried more of the immediate caregiving demands due to logistical constraints and how he actively sought to compensate by taking on other responsibilities:

*The burden just has a way of shifting to my wife … if someone is immediately ill or needs to be picked up … I’m forty-five minutes away. She’s at home. So, then I try to make up for that. By taking on other responsibilities, right, whether it’s taking the kids to the programs in the evening or dinner or clean up or whatnot. (P14, Father)*

Mothers often described shouldering the “heavier lifting” of caregiving, particularly in medical and daily personal care tasks. Several mothers expressed a sense of gendered caregiving expectations, where they were the primary point of contact for medical care and emotional support, while fathers assisted in logistical tasks such as transportation.

*She relies more on me as opposed to her dad and her brother for that stuff like, washing her hair, because I am a lady …. Typically, ninety percent of the appointments are me and her. Because, again, I am the mom at home, and I’m the one that knows …. Dad drives and pushes around the wheelchair during the appointments. But it’s usually the other heavy lifting that lands on me. (P9, Mother)*

Yet, despite these imbalances, both fathers and mothers carried the emotional labour of caregiving, providing comfort, understanding, and empathic support to their children, highlighting the interconnectedness of well-being within family systems. Parents found small but meaningful ways to connect with their child through their pain, highlighting their shared emotional investment in caregiving.

*There were times when, even through the pain, just driving up the road to get a smile from her was enough. We’d go to Wendy’s, and she’d look forward to that little break. Even now, she remembers it. It’s small, but those moments became our way of coping together. (P5, Father)*

For many caregivers, the weight of witnessing their child’s suffering was profound. Parents described feeling powerless in the face of their child’s chronic pain, yet deeply committed to offering what comfort they could.

*We share ups and downs. Sometimes I believe I can do it, then feel like I don’t know, just so helpless. It’s the worst because you can’t take [the pain] away, like you can’t. But you can try to understand, you can help her. You can ease it, but it never goes away. (P6, Mother)*

Across multiple accounts, mothers and fathers described the dual reality of caregiving, navigating both the physical and logistical burdens while also bearing the emotional toll. While the division of labour was often unequal, caregiving was also a deeply shared experience, marked by moments of exhaustion, helplessness, and quiet connection.

Coupled caregivers described how their relationships with their partners became strained under the weight of caregiving. Many spoke about the need for teamwork to manage caregiving demands while also recognising the challenges in maintaining their spousal relationships.

*It’s definitely like you need two people to do this. I can see why parents would split up because if you feel like you can’t get support from your partner … but it takes so much energy with her, there’s just no way I could be doing this alone. (P20, Mother)*

For some, caregiving led to emotional disconnection within their relationship, as their child’s needs took priority over their own connection as a couple. While this all-in focus on caregiving was often framed as temporary, many expressed hopes that they could eventually return to their relationship with renewed focus once their child’s condition was more manageable.

*It is impacting [partner] and my relationship. It’s almost like we said the other day: it’s almost like our relationship is on hold because we’re both giving so much that we don’t have time to be with each other. We’re just a partnership working together to try and get through this right now which is quite hard. But at the same time, at least we’re not separating or breaking apart; we just accept it. It is what it is, and we don’t necessarily work on or build our relationship. We work on and build our children. (P24, Father)*

*So, we tried as much to be like, we’re in this together. This is what families do, but at the end of the day, of course, there is an impact on our relationship. (P16, Mother)*

While coupled caregivers leaned on their partners for support, despite the strain it placed on their relationships, single caregivers navigated caregiving without the help of a co-parent. Their roles were multifaceted: they served as primary caregivers, financial providers, and emotional supporters. Many described feeling intense stress, as they often had to prioritize their child’s needs over their own health and well-being, sometimes leading to burnout.

*The hardest part of being a single parent is having no one to lean on, no one to hand the baton to when you’re at your limit. You’re the last line of defense, and sometimes it feels like you’re fighting battles no one else can see. (P10, Mother)*

For single parents who did not have an engaged co-parent, the burden was particularly heavy. Many relied on extended family, especially their own parents, to help manage caregiving responsibilities.

*As a divorced mother, it was all on my shoulders because her father was just not able to really assume any of the responsibilities when it came to our healthcare. So, I had to do all of those things, the planning, the driving, the conversations, I had to do all that. (P25, Mother)*

Although caregiving can strain relationships, caregivers shared that spending more time supporting their child led to deeper emotional bonds and created a unique closeness that may not have been possible otherwise. Many described their caregiving as both a responsibility and a privilege; some expressed that the experience had profoundly shaped their relationship with their child.

*I am a lot closer to my daughter, closer than I could have ever been … Actually, it was even more so when [the pain] was more severe. I don’t, I don’t think I would have been as close to my child at all if it weren’t for all of this. (P32, Father)*

Caregivers found meaning in small yet significant moments of connection, highlighting how caregiving, despite its challenges, became an opportunity to deepen relationships and form bonds. This benefit finding was important as caregivers reflected on how, despite the hardship, their experiences created stronger emotional ties and lasting memories with their child.

*We were, you know, I was able to really adjust my work schedule in a way that made me able to participate with him as much as I felt like I needed to. And it’s it sounds like ridiculous, but there is something kind of sacred about this time. We spent an incredible amount of time together. Like, because we would drive to [healthcare organization] three days a week. Like you know, he’s a thirteen-year-old dude sitting in a car, just chit-chatting with him is like something that I will not take for granted. We actually then we would always do, do physio together, like I would always sit with him. (P16, Mother)*

However, the deepened bond with the child in pain sometimes created distance from other siblings. Caregivers described how they struggled to balance their attention between their child with chronic pain and other children. This often brought up guilt and inadequacy.

*I felt that I was somehow cheating [older sibling] of my time, but she needed more attention, and that was hard. I knew it was hard for him [sibling], too, and I struggled with that balance every day. (P7, Mother)*

Some caregivers found ways to involve siblings in caregiving moments, though they later reflected on whether this was enough to meet their children’s emotional needs.

*We did our best, but in the moment, it was hard to meet both kids’ needs because they were so different. His sibling would sometimes join in with his therapies, like when he had to touch different places on the ground. His younger brother would say, ‘Touch my belly button, touch my nose,’ trying to help. They became really close, but I realize now we focused so much on [the child in pain] that maybe we could have done more for his brother, too. (P14, Father)*

For many caregivers, the daily demands of caregiving, unpredictability of their child’s condition, and emotional toll made it difficult to maintain friendships. They described feeling lonely and isolated as they withdrew from social interactions, sometimes because of time constraints, but also because they felt misunderstood.

*I guess the thing it impacts the most is my own social life. Like I’ve really lost, I haven’t lost friends, but I haven’t seen friends for quite a while because I was always the friend that would reach out and arrange a catch-up and they kind of relied on me doing that and I’m not doing that at the moment and so I’ve noticed that I’m not hearing from them either, so I feel I’m feeling a bit isolated socially. (P23, Mother)*

*Our circle and our world and my world has also become a lot smaller … our world basically revolves around hospitals and doctors. (P27, Mother)*

Beyond the logistical challenges, caregivers also expressed nonverbal but deep invalidation from friends and social circles. Many felt that friends who were unfamiliar with youth chronic pain struggled to grasp the depth of their experiences.

*With my health and my daughters, sometimes you have to cancel and sometimes, people aren’t understanding of that. So, you have sort of your close group who understands. Then your extended group, some of them get it, some don’t. I’m at peace with that now. But it’s hard when you know people just stop asking you to do stuff because you have to cancel sometimes. (P8, Mother)*

*I feel like with friends, it’s too complicated for people to understand. Early on, when she was really young and we were trying to explain because we were trying to figure it out ourselves, you can almost see peoples’ eyes glaze over. (P13, Father)*

Some caregivers also note that friendships became centred around their child’s illness, making it difficult to reconnect with their own identity.

*It’s just like, you know, sometimes it’s hard because it’s like that almost becomes the focus, when you don’t want it to be. You want to go out to escape everything and be a person again. And like for [friends], they’re just like, ‘hey how is she, how’s the family? How’s your kid? How’s, how’s all this going with the pain and stuff?” So sometimes it’s good to see other people, but sometimes it is more like you’re just really, re-living what you’re living. (P11, Mother)*

*Sometimes you don’t really want to or feel like you need to get into all the details with your friends … We’ve got good people around us who generally understand in most cases like our like employers, but our immediate friends aren’t generally in the know and this sort of stuff. (P14, Father)*

Many caregivers spoke about how caregiving tested their friendships, revealing which relationships were truly supportive. Some found that people they expected to be there drifted away, while others unexpectedly stepped up.

*Well, it was interesting. So, when she got diagnosed. The people that we had, certain people that we were around, that we thought were really good friends, they kind of disappeared, and then we had people that we really didn’t have a huge connection with; they came out of the woodwork. Those are our deepest relationships now. It’s like people rose to the surface, and then people like disappeared. (P6, Mother)*

*But you know, there’s those few people that do check in, but you do definitely find the social circles flip. And change quite a bit. We’ve lost family and friends. We have family that are like, they are there but they’re not. They’re there to come over for dinner, but not bring her dinner, you know. (P9, Mother)*

At the same time, caregivers actively sought new friendships to help manage their loneliness, particularly with other caregivers. Peer support became a vital resource, offering practical help and emotional support.

*Some of my best connections are through some of these moms that I’ve met and being able to reach out to them to ask them, ‘have you ever gone through this?’ … just being able to learn about it. But also, being able to share what you’re learning helps because I’ve helped quite a few moms. (P9, Mother)*

For some, community support, provided through faith-based groups, online spaces, or local networks, served as an essential protective buffer against isolation.

*In church, we pray for my daughters, and we have a group that sometimes helps, too, right? Because if I’m taking her to a hospital, or whatever, its’s hard. So, they help me, help us, they take care of my other kids, and stuff like that. (P21, Father)*

*It was nice to have somebody that has been going through you know, very similar things … It’s comforting, to be honest. (P15, Mother)*

*I know of private Facebook groups … just reading other people’s posts … helps me feel like I’m not alone, it can help a bit with feeling less isolated. (P18, Mother)*

Despite the social challenges, caregivers overwhelmingly emphasized that social support, especially from those who truly understood, was crucial in helping them navigate the emotional burden of caregiving.

### 3.2. Loss and Neglect of Personhood

Caregivers described two layers of self-sacrifice: unconscious sacrifice and conscious prioritization. Both types of self-neglect created a cumulative toll, but caregivers persisted in their sacrifices because of their ingrained love and responsibility for their children. Many caregivers described how they experienced a pattern of self-neglect without fully realizing it.

*Because nobody asked me, I don’t know if I ever thought about it. I don’t know if I ever even thought about myself at all. And I certainly didn’t think [about taking care of myself]. (P5, Father)*

*At some point, I just switched gears and put myself on hold … It’s become so automatic that I don’t even know what I need now. (P17, Mother)*

Caregivers poured their time, attention, and energy into their child’s medical needs, school advocacy, and daily care, gradually setting aside their own well-being. Many only later recognized the physical and emotional toll of their self-neglect.

*I just couldn’t even think about going to the doctor for myself. I was so focused on managing his needs, and my own health just kind of faded into the background. (P22, Mother)*

*I could have gone to physical therapy, but then I’d miss his appointments, so I put it off … for years. I only realized later how much I had neglected my own body. (P5, Father)*

Some caregivers were acutely aware of the sacrifices they made, yet still chose to prioritize their child’s needs above their own, seeing it as an inevitable part of caregiving.

*I think I stopped thinking about what I needed a long time ago. It’s like my feelings don’t even register sometimes. There’s no time or space for them. (P11, Mother)*

*I’ve decided my role is to keep things going for him, even if it means I go without things I need. That’s just how it has to be. (P13, Father)*

Many caregivers brushed aside physical symptoms such as their own chronic pain or exhaustion because they saw their self-sacrifice as necessary. Even though they recognized the long-term consequences, they felt unable to prioritize their own health.

*I know I should address my back pain, but I consciously ignore it because I can’t afford to be unavailable. I can’t let the family routine fall apart. (P2, Mother)*

*I know it’s draining me, but it feels like the only way to make sure he has what he needs. It’s a choice I make to put my energy into him, not into myself. (P19, Mother)*

However, caregivers also acknowledged the cost of their self-neglect, not only for themselves but also for their ability to care for their child.

*That adds to the stress. And so, it doesn’t actually leave me much time to relax, which then loops back to not being the best father that I can be to either of the kids. (P24, Father)*

*I wish I had reached out sooner to my doctor to get that support for myself so that I’m better able to support my child, and be a better parent. (P1, Mother)*

Many caregivers transitioned from full-time careers to part-time work or became full-time caregivers. While some were able to adjust their work schedules to accommodate caregiving demands, others experienced a profound shift in identity, leading to grief, isolation, and a sense of losing a core part of themselves.

*When I left my employment, I left all my friends behind. Part of it was intentional in the sense that I felt like I was being re-traumatized every time I talked to somebody. The other part of it was like I so desperately wanted to be back there that it just made me feel really jealous and angry. So, I think the biggest struggle it’s just feeling really lonely, in the sense that, like I don’t have a lot of peers my own age to socialize with. (P4, Mother)*

*I used to get such a sense of achievement at work, you know, through my job, and now I don’t get much of that anymore. (P18, Mother)*

Other caregivers were able to retain some professional engagement, particularly if they had remote work or flexible job arrangements. However, even when flexibility was possible, the strain of balancing work and caregiving remained significant.

*We both are lucky to have a degree of flexibility [in our work]. But it’s hard because we both missed time from work due to this, so it affects us quite a bit in that regard, and naturally, because I usually have to be at work in person, whereas [my wife] doesn’t, she has to be more flexible. (P14, Father)*

*So, work-wise I am incredibly lucky that I have a really flexible job, like so I was able to really move my schedule around his appointments. (P16, Mother)*

Caregivers described how self-sacrifice, whether unconscious or intentional, became ingrained in their daily lives. Over time, their own needs faded into the background, contributing to both physical and emotional exhaustion. The cumulative toll of self-neglect, career loss, and financial instability left many caregivers struggling to maintain their sense of self.

**Theme 2: Deep Interrelatedness and Blurred Boundaries**

This theme reflects the intertwined well-being of both youth and caregivers, emphasizing a family-centred approach to youth chronic pain. Caregivers shared how the journey to wellness for families relies on validating and addressing the interconnected health of both caregivers and their children. They described how validating children’s experiences of pain, ensuring access to integrated, personalized care, and prioritizing caregivers’ own health reinforced resilience and holistic well-being within the family. This interwoven dynamic shaped how caregivers understood their role, their self-sacrifices, and their own self-investment.

### 3.3. Permission for Self-Investment

Building on earlier descriptions of profound self-neglect and identity loss, this subtheme captures caregivers’ tentative shift toward recognizing the importance of their own well-being—though often constrained by guilt, systemic barriers, and the perceived primacy of their child’s needs. Before they could meaningfully prioritize their own health and self-care, caregivers expressed their need for assurance that their child has access to timely, integrated, and holistic care. When caregivers felt secure that their child’s physical, emotional, and social needs were met, it created space for them to step back, reflect on their own well-being, and invest in self-care without guilt or anxiety.

*So, yeah, if you asked me now, like, what else could help? I’m trying to think of anything to help her mentally … if she was OK mentally, I was OK mentally. (P5, Father)*

*Self-care, if you have a child who is not safe, doesn’t rate … It feels not important. We know it is, but it rates down the hierarchy of needs so … we have to be very intentional, I guess, to bring it to the parents’ needs and tie it to the child’s wellness. (P10, Mother)*

Caregivers recognized that prioritizing their own well-being was crucial to sustaining their ability to care for their children and families. However, many described a persistent internal struggle to give themselves permission for self-care without guilt.

*Giving myself permission to be a priority too … That would be what I would need to do. So, being able to be like, maybe [my child] needs something, and I still need to go out for a walk, and you’re going to have to wait. That’s … but that’s my own internal stuff—having to give myself permission. (P2, Mother)*

*For me, I know that I should take better care of myself and that I … I should get moving more, and that’s important. To care for yourself … I think this caregiver course that I took, I wish I’d taken that sooner. (P1, Mother)*

At the same time, caregivers reflected on the consequences of prolonged self-neglect. Some expressed regret for not prioritizing their physical and mental well-being earlier, only realizing the impact when their own health had deteriorated.

*I think I’m going back to that one about just like making sure that people are taking time for themselves, that it’s so important. And I’ve heard it over and over … but I’ve never done it until this year, and it’s like, it almost feels too late for some things, right? Like you let things go so far, and then you’re like, oh, God, what did I do? (P4, Mother)*

*I’m getting older now, and I know that my health, my ability to help my child—if I don’t stay healthy, that’s going to impact her negatively. So that’s sort of the motivation for me to just … I’ve got to, you know, I’ll take walks. I’ve got … my mental health will affect her as well as my son. (P12, Mother)*

Caregivers identified key resources that would help them sustain their own well-being, including mental health support, exercise, peer support, respite, and financial or household assistance. Many emphasized the need for accessible mental health resources tailored for parents managing youth chronic pain.

*Hands down, therapy, having a one-to-one therapist that I can see and access on a regular basis has been critical …. My hope for the world is that there’d be accessible therapy or resources for kids and parents. (P2, Mother)*

*I’ve also seen a psychologist … just have a space and a place to present and process everything else, that’s so important. (P28, Mother)*

*It’s just that external perspective from therapy, without the emotion of a family involved, that has been super helpful because it’s brought me that understanding which enables me to then see the impacts of my actions and how to change them. (P24, Father)*

However, caregivers stressed the importance of broadening available resources beyond psychotherapy to include peer support, respite care, and funding for wellness activities.

*Yeah, it’s very validating to know that you’re not alone. And other people understand and are going through the same thing. (P8, Mother)*

*Knowing that [other parents] go through the same kind of anguish … having someone else to talk to, knowing that you’re not alone in life, it makes a big difference. (P27, Mother)*

Caregivers described respite care not just as a means of relieving stress, but as an opportunity for meaningful self-investment. They suggested funding activities that promote caregiver well-being, such as exercise programs, household assistance, and opportunities to take a break from caregiving duties.

*I think in a perfect world, I would have the funding from the government to have someone come into the house for a couple of hours a day and be with [child’s name] and let me go out and go to the gym or go for a coffee with a friend or something like that. I’d need some respite from like they would be the biggest thing. (P23, Mother)*

*… if someone said ‘hey, you know, you get you have access to, like so many visits to the gym’ or whatever, one hundred percent I would do it. (P6, Mother)*

*We’re talking to [government organization] about funding … to have somebody help transport and, you know, come into the house potentially so that [my partner] and I can go and do things. (P24, Father)*

*I think if I had more support in terms of respite care, I would feel less overwhelmed. Right now, it feels like there’s no break at all. (P13, Father)*

A recurring theme in caregivers’ narratives was the powerful protective role of social support. Caregivers who had peer support networks, social validation, or structured respite resources reported feeling more equipped to handle the demands of caregiving.

*When you’re in a support group, you feel seen. It’s a place where you can share your feelings without judgment, and that’s so important for healing. (P2, Mother)*

*Talking to others who get it helps me feel like I’m not alone. We share tips and tricks, and that sense of community is just so comforting. (P4, Mother)*

*The group really feels like a family. We celebrate the wins together, and when things are tough, we’re there for each other. That support is invaluable. (P5, Father)*

Caregivers’ experiences highlight the interdependence between their well-being and their child’s health. While many caregivers struggled to give themselves permission for self-care, they also recognized that prioritizing their own mental and physical health was essential, not only for themselves but for their child and family. Access to mental health resources, peer support, respite care, and practical assistance were identified as critical components in fostering caregiver resilience. Caregivers emphasized that self-investment should not be seen as a luxury but as an integral part of sustaining their caregiving role.

## 4. Discussion

The profound impacts of youth chronic pain extend into all aspects of family life [10,41,42], creating an ongoing struggle for caregivers to balance their responsibilities with personal well-being. These findings highlight how caregiving strains emotional bonds between partners, disrupts sibling relationships, and fractures social connections, leaving caregivers isolated and overwhelmed. Despite these challenges, caregivers show remarkable resilience by adapting roles, redefining identities, and continuing to support their families.

This study underscores the interconnectedness of caregiver and child well-being. Caregivers frequently prioritize their child’s needs above their own, sacrifices felt more deeply by those with fewer supports. Participants identified early access to mental health services, tailored educational resources, and peer networks as essential for fostering resilience. Addressing these inequities requires systemic approaches that acknowledge the differing caregiving burdens and empower families navigating youth chronic pain [43]. While this study focused on shared caregiving experiences, future analyses should explore how intersecting factors such as race, rurality, and gender identity further shape these experiences. An intersectional lens may reveal layered inequities that impact access to support and well-being.

Notably, our findings challenge traditional gendered caregiver narratives. Fathers actively embraced day-to-day caregiving, disrupting conventional expectations that historically confined men to financial provision roles [44,45]. This evolution mirrors broader cultural shifts that are increasingly recognized in research [46,47,48,49]. However, gender disparities remain evident [50,51]. Mothers continued to bear the bulk of caregiving, particularly in medical management and daily care. Although fathers expressed efforts to compensate, mothers still carried the “heavier lifting” shaped by entrenched gender expectations and logistical barriers. These findings reinforce the need to critically examine how caregiving roles are negotiated within families [52]. Although fathers’ increased involvement marks progress, it also introduced new concerns about how male caregivers manage distress in the context of societal expectations and masculinity norms [53,54,55]. At the same time, mothers continue to bear the cumulative emotional and physical toll of caregiving. Targeted and accessible interventions are needed to support caregivers of all genders. For fathers, interventions should include coping strategies that validate caregiving while challenging outdated gender norms. For mothers, support must address persistent structural inequities that place disproportionate burden on them. Traditionally, caregiving has been idealized as an act of self-sacrifice [56,57], disproportionately carried out by women and shaped by cultural expectations that prioritize children’s well-being over caregivers’ needs [58]. Our findings show how these ingrained norms continue to influence caregivers; many felt guilt and described self-care as selfish or undeserved. This aligns with prior research showing that caregivers often experience internal conflicts about prioritizing their own needs [2,4,59], compounded by limited time, support, and resources. However, our study also revealed a growing awareness among caregivers about the direct link between their own well-being and their ability to care for their child. Caregivers increasingly recognize that their physical and mental health affects their family relationships and child’s recovery. Despite this awareness, few structured, accessible caregiver support options exist in pediatric pain care [8,45]. Addressing this gap requires a systemic shift from caregiving as self-sacrifice to caregiving as a shared responsibility that values and invests in caregiver health.

Yet, caregivers face many barriers to acting on this recognition—including limited access to respite care, financial assistance, mental health support, and time [5,6]. Social isolation compounds these challenges, making it even harder to prioritize self-care [38]. To address this, care models must invest in caregiver health through tangible supports and community-based networks. Virtual care offers a promising path forward. Online educational programs can build caregiver knowledge and normalize self-care as an essential aspect of family functioning [60,61]. App-based CBT, tele-psychotherapy, and peer mentorship programs can help caregivers manage stress and feel less alone [62,63,64,65]. Digital solutions that prioritize caregiver well-being equip families to navigate chronic pain with greater resilience. By combining systemic change with individualized tools, we can reframe caregiving as a role that honors the health of both the caregiver and the child. The ripple effects of youth chronic pain extend beyond parent–child relationships. Caregivers expressed guilt and distress about unintentionally neglecting siblings, whose routines and roles were often upended. These shifts, missed activities, increased expectations for independence, and disrupted family rhythms strained sibling bonds and left caregivers feeling torn. This echoes previous findings and underscores the need for family-focused interventions that rebuild connection and inclusion across all family members [66,67,68,69]. Virtual platforms offer an accessible means to support siblings, facilitate shared experiences, and foster resilience despite ongoing caregiving demands [9,70]. This study also demonstrated the value of authentic caregiver engagement. By involving caregiver partners as interview co-hosts and co-authors, we fostered a more trusting, relatable research space. This led to deeper participant insights and reinforced the importance of patient involvement in designing meaningful solutions [71].

### Strengths and Limitations

This is the first study to explore the mental and physical health needs of caregivers of youth with chronic pain. A key strength was the strategic recruitment of a diverse sample, capturing a range of identities, caregiving roles, and healthcare system experiences. Including participants from both Canada and Australia enhanced transferability across differing systems and cultural contexts [12]. Our rigorous thematic analysis [34], supported by lived experience partner engagement, yielded well-grounded findings and enriched interpretation [40]. Co-hosted interviews created a trusting, participant-centred space that encouraged honest, reflective dialogue. Additionally, the study offered emerging insights into evolving familial dynamics—particularly the changing role of male caregivers. However, several limitations highlight future directions. Non-English-speaking caregivers were excluded, limiting representation of linguistically diverse perspectives. The exclusion of caregivers with untreated psychiatric illness may have omitted those experiencing heightened psychological vulnerability. Although this study captured valuable perspectives from male caregivers, their numbers were limited. Future research should purposefully engage underrepresented caregivers, including LGBTQ+, Indigenous, and digitally underserved populations. While the online interview format enabled broad geographical reach, it may have excluded participants with limited digital access. Lastly, transcripts were not returned to participants in alignment with naturalistic inquiry principles [22], ensuring authentic capture of first-impression narratives without prolonged engagement [28].

## 5. Conclusions

Youth chronic pain reshapes family life, straining caregiver relationships and overwhelming daily functioning. Caregivers in this study reported feeling isolated, exhausted, and unsupported—but also showed resilience, adaptability, and a growing awareness of the need to care for themselves as well as their children. These findings signal an urgent call to embed caregiver well-being within pediatric pain care. Mental health support, peer networks, and tailored education must be made accessible through equitable, systemic strategies. Virtual interventions—such as app-based CBT and teletherapy—can empower caregivers to manage stress and sustain their roles. By integrating these tools into caregiver-focused platforms, we can promote long-term resilience for families navigating chronic pain. Future research should focus on piloting and evaluating the PoPPc digital hub and actively recruiting underrepresented caregiver groups, including those who are non-English-speaking, rural, or digitally excluded.

## Figures and Tables

**Table 1:** Participant characteristics.

Characteristic	Value Number of Participants (n = )
**Primary Caregiver Gender, n (%)**	
** Region: Australia** Female	**n = 12** 6 (50.0%)
Male	6 (50.0%)
** Region: Canada**	**n = 20**
Female	15 (75.0%)
Male	5 (25.0%)
**Primary Caregiver Sex Assigned at Birth, n (%)**	
** Region: Australia**	
Female	6 (50.0%)
Male	6 (50.0%)
** Region: Canada**	
Female	14 (70.0%)
Male	5 (25.0%)
	*1 participant did not specify*
**Age (y)**	
** Region: Australia**	
Primary Caregivers (35–55)	Mean: 49.5
Primary Caregiver’s Children	Mean 14.2
** Region: Canada**	
Primary Caregivers (30–59)	Mean: 48.1
Primary Caregiver’s Children (11–21)	Mean: 15
**Ethnic Origin, n (%)**	
** Region: Australia**	**n = 12**
Aboriginal/First Nations	1 (8.3%)
Black (e.g., African, Haitian, Jamaican, Somali)	1 (8.3%)
European	4 (33.3%)
White	2 (16.7%)
Prefer not to answer Mixed race	1 (8.3%) 3 (25.0%)
** Region: Canada**	**n = 20**
Black (e.g., African, Haitian, Jamaican, Somali)	1 (5.0%)
Chinese	2 (10.0%)
European	6 (30.0%)
South Asian (e.g., Afghanistan, Bangladesh, Bhutan, India, Maldives, Nepal, Pakistan, Sri Lanka)	1 (5.0%)
White	8 (40.0%)
Other (Specified: Canadian of European Descent)	1 (5.0%)
Other (Specified: Mixed race Eurasian)	1 (5.0%)
**Highest Level of Education Completed, n (%)**	
** Region: Australia**	**n = 12**
Secondary school College, technical school, or TAFE	2 (16.7%) 1 (8.3%)
University	3 (25.0%)
Post-graduate degree	5 (41.7%)
Did not respond	1 (8.3%)
** Region: Canada**	**n = 20**
Secondary school College, technical school, CEGEP	1 (5.0%) 4 (20.0%)
University	9 (45.0%)
Post-graduate degree	5 (25.0%)
Prefer not to answer	1 (5.0%)
**Estimated Total Annual Household Income, n (%)**	**AUS n= 12**
(before taxes, including all household members)	**CAD n = 20**
** Very Low:**	
<$29,000	1 CAD (5.0%)
** Low:**	
<$30,000–$59,000 AUD	1 (8.3%)
<$30,000–$59,000 CAD	1 (5.0%)
** Medium:**	
$60,000–$89,999 AUD	1 (8.3%)
$60,000–$89,999 CAD	2 (10.0%)
** High:**	
>$90,000 AUD	6 (50.0%)
>$90,000 CAD	16 (80.0%)
** Prefer not to answer:**	1 AUS (5.0%)
	1 CAD (5.0%)
**Marital Status, n (%)**	
** Region: Australia**	**n = 12**
Married or common law	9 (75.0%)
Separated or divorced	1 (8.3%)
Prefer not to answer	2 (16.7%)
** Region: Canada**	**n = 20**
Married or common law	18 (90.0%)
Single	1 (5.0%)
Prefer not to answer	1 (5.0%)
**Current Living Situation**	
** Region: Australia**	**n = 12**
Spouse, Children	4 (33.3%)
Children	4 (33.3%)
Spouse, Children, Parent(s)	2 (16.7%)
Prefer not to say	2 (16.7%)
** Region: Canada**	**n = 20**
Spouse, Children	18 (90.0%)
Children	1 (5.0%)
Spouse, Children, Parent(s)	1 (5.0%)
**Duration of Chronic Pain, n (%)**	
** Region: Australia**	**n = 12**
<12 months	3 (25.0%)
1–3 years	3 (25.0%)
>3 years	6 (50.0%)
** Region: Canada**	**n = 20**
<12 months	3 (15.0%)
1–3 years	4 (20.0%)
>3 years	13 (65.0%)
*Participants asked to describe duration of child’s pain in months or years.*	
**Type of Chronic Pain Experienced by Child, n (%)**	
** Region: Australia**	**n = 12**
Neuropathic & Nociplastic Pain Conditions	3 (25%)
Headaches & Neurological Pain	2 (16.7%)
Abdominal & Pelvic Pain	2 (16.7%)
Musculoskeletal & Joint Pain	3 (25.0%)
Widespread Pain & Multi-Site Conditions	2 (16.7%)
** Region: Canada**	**n = 20**
Neuropathic & Nociplastic Pain Conditions	4 (20.0%)
Headaches & Neurological Pain	1 (5.0%)
Abdominal & Pelvic Pain	4 (20.0%)
Regional & Limb Pain	2 (10.0%)
Musculoskeletal & Joint Pain	9 (45.0%)

**Table 2:** Qualitative results.

Theme	Sub-Theme	Example Narratives
1. Bearing the Weight and Sacrifice of Caregiving	1.1 Redefined Relationships and Roles	Life’s taken a full like one eighty. I’m now a full-time caregiver. I don’t always have my partner to be talking back and forth. From when I get up, I’m on the go with her; when she’s up, I’m up. (P11, Mother) I am not always the best father that I can be to either of the kids because I am just pulled. I need to work really well and deliver at work in order to have the financial security we need to have all of the opportunities that we need for [child’s name] to get to her appointments, to try new things, to see new doctors, to see new specialists. So it hasn’t got in the way of my work, but it I think it gets in the way of me being as good as I could be at home because of the overall load (P24, Father) I suppose my wife is so good at it [day-to-day caregiving]. I stay in my lane and work with my strengths, which sometimes is just making the kids laugh … taking them out, trying to make them happy in the short term and in the long term, too. But we both have our ways of helping them cope. (P14, Father) All my emotions are through my child; she is everything. So, if she’s sad, I’m sad. If she’s hurt. So, my attachment to my child is very serious. (P15, Mother) In terms of the relationship between my wife and I, it has really been impacted. It’s kind of for us, obviously; the priority is very clear. It’s our child. There is no animosity towards each other about how we’re spending our time. If I could spend my time in any way that [my wife] would appreciate, it’s with her kids and me and vice versa. So, there’s no hard feelings about our relationship. (P5, Father) I was always aware, from an early age, that I couldn’t ignore the sibling. When I had pockets of time or energy, I tried to give that to him. It was hard, but I never wanted him to feel forgotten. (P12, Mother) [Sibling] is affected because he feels like he needs to step up to reduce the stress. So, I have to be very careful that I don’t ask too much because he will just do in order to try and fill the gap. He’s very empathic. It is impacting our relationship. (P24, Father) Some of our family didn’t understand it, especially at the beginning, and I felt angry that some of my family were not getting this. (P16, Mother) Like, I don’t know when you say it out loud to other people. It’s just like because it’s just your normal and they hear it. And they’re like, ‘oh, wow, I didn’t realize’ … so it’s tricky. (P25, Mother)
	1.2 Loss and Neglect of Personhood	I don’t even have my own space anymore. My work, my time, it all just blends into what they need, and I guess I’ve just gotten used to it. (P8, Mother) Sometimes we don’t take the time to kind of go, ‘Oh. I am feeling like overwhelmed.’ (P6, Mother) I am always on, you know? Ready for anything he needs. I didn’t notice how exhausting it was until someone else pointed it out. (P12, Mother) I put everything, like my heart and soul, into caring for [child’s name], and trying to hold it together … I know full well that I have neglected my own needs. (P18, Mother) I just ignore the aches and pains now … they’re always there, but I don’t even register them because it feels like there’s just no time to deal with them. (P25, Mother) I’d rather spend that money on his therapies than on something for myself. It’s not even a question; he comes first. (P10, Mother) My kids weren’t suited to go to after-school care or anything like that, so I restricted my working hours a lot, and I certainly restricted my social activities. My boys really got my focus so for a lot of years, I did end up quite isolated. (P10, Mother) I think one of the greatest things that happened to my daughter was … the first thing out of the pediatrician’s mouth was: ‘I believe you.’ So that set the trajectory … let’s validate the pain as a first step. (P12, Mother)
2. Deep Interrelatedness and Blurred Boundaries	2.1 Permission for Self-Investment	It would have been nice to be like, ‘hey, this is an option’ … to have them do like a mini counseling session. (P6, Mother)
		The parent component is probably the most valuable. Just having that reassurance that you’re not alone and you’ve got other people going through it. Even though we didn’t go to formal counseling, and I know I have people I can talk to. (P6, Mother) It’s just a really a question of empathy and understanding. What’s really important at that time is not really stuff like advice, or it’s just to be there really. (P5, Father) Finding a housekeeper who will come and be consistent would be great. Especially if it is covered through [program]. (P9, Mother) Fortunately, when I applied for a Carer Card, I was told that I also qualify for some home help once a week … that was a big help because I find time gets away from me. (P27, Mother)

## Data Availability

The data presented in this study are not publicly available due to ethical and privacy restrictions. Participants did not provide consent for their full interview transcripts to be shared beyond the research team. De-identified excerpts supporting the findings may be available from the corresponding author upon reasonable request and with appropriate ethical approval.

## Supplementary Materials

The following supporting information can be downloaded at: https://www.mdpi.com/article/10.3390/children12070911/s1.
