# Supplementary material for: Balancing Care and Sacrifice: Lived Experiences and Support Needs of Primary Caregivers in Pediatric Chronic Pain Across Canada and Australia
Source: Children (Basel). 2025 Jul 10;12(7):911. doi: 10.3390/children12070911 (PMC12293230; doi:10.3390/children12070911)
Supplement: Supplementary file 1 [file children-12-00911-s001.zip › children-3710396-supplementary.pdf]

Parent Interview Guide

Instructions for the interviewer:

- The first question of the interview guide must always be asked first. It is a general question that will include different interview themes. The order in which the following questions will be asked can be adapted as a function of what the participant is describing and the order in which s/he volunteers the information.
- Never cut off a participant. Always let the participant speak even if s/he is anticipating another question. In this case, it might be pertinent to ask that question earlier than suggested in the interview guide.
- If a participant's narrative becomes tangential, do not cut off the person but rather reorient him/her toward an interview question when the opportunity presents itself (e.g., when s/he mentions something that could be related to an interview theme, or when there is a silence).
- Always ask open-ended questions. Do not suggest answers (use probes only when topics are not spontaneously being discussed).
- Do not hesitate to follow-up on a question you have asked or to reformulate a question if someone believes they have nothing to say about a specific topic.
- Give enough time for the person to think and reflect so that they can answer the questions. Do not try to fill in the silences.
- Try to anchor as much as possible the interview in specific lived experiences. Try to avoid questions that are too general and that are not directly related to the daily experiences of participants.
- Use terminology that participants are most familiar with. Avoid as much as possible scientific terminology, unless someone uses it spontaneously. For example, you can use coronavirus or COVID-19 depending on the term the participant is most familiar with and uses (same for "epidemic / "pandemic")

Introduction:

Thank you for making time to talk with me today. As some background, we know that parents of children with chronic pain (CP) may experience high stress and poor physical and mental health. Many are also impacted in their family relationships and parenting. When parents face these challenges, their child with CP is more likely to have worse pain, and their families suffer. So, it is critical to have interventions specifically for parents to support their well-being, as well as the quality of life of their children and families. The online platform, *Power over Pain (PoP)* currently helps youth with CP get the right treatment at the right time. but we know there is not enough information in it yet specifically to support parents and caregivers. This research project is focused on identifying interventions to include in the youth PoP portal that addresses the well-being and needs of parents of children with CP.

The goal of this interview is to understand what your needs are as a parent caring for a child with CP. I will ask you some questions so that you can tell me details about your current experience and any resources that you think would be helpful to support your own health, and well-being and parenting needs. The interview will be audio recorded and kept private. Your name or any other identifying information will not be included during the recording, except

your voice. Participating or withdrawing from the study will not have any effect on the care your family will receive. You can stop the interview and withdraw from the study at any point. You may contact the Research Coordinator to request your interview data be removed from the study before analysis begins. Please let me know if a question makes you feel uncomfortable or if you would like to take a break.

As was shared with you in the study information, there is an adult version of the Power over Pain portal. Hopefully you have had a chance to look at the resources that are available on both PoP portals.

Thank you for consenting to this interview, and to having the interview recorded for the primary purpose of creating a transcript of the conversation. Are you comfortable to start the interview now?

Questions:

1. **Can you tell me about your child's life living with CP?**

   Probes:
   - How long has your child been living with CP?
   - Tell me about how your child's pain has impacted their mental health (e.g. sleep, mood, behaviour)
   - How has your child's pain and mental health impacted their life and relationships (e.g. school, friendships, family relationships, recreational activities)

2. **Can you tell me about your life as a parent of a child(ren) with CP?**

   Probes:
   - Tell me about your mental health challenges parenting a child with CP (e.g. sleep, mood, behaviour etc.)
   - How has caring for your child impacted your role(s) and relationship(s) (e.g., employment/work, parenting/carer, spouse relationship, family/sibling relationships, friendships/peers)?
   - How do you talk to your child about their pain?
     - What challenges do you have talking to your child about their pain?
     - What makes it easier to talk to your child about their pain?
   - Do you have pain yourself?
     - If yes, can you tell me about your experience living with CP
     - Tell me about how living with pain has impacted parenting your child

3. **What are the biggest challenges you face in your role(s) as a parent(s) of a child with CP?**

   Probes:
   - What is something that you know now that you wished had learned earlier in your child's diagnosis/experience with CP?

4. **What are your biggest strengths in your role as a parent(s) of a child with CP?**

5. **What has helped you in your role as a parent of a child with CP?**
   Probes:
   - What types of online information/resources/apps have you found helpful? (e.g., pain education, carer role/parenting education, resource access for families/friends, symptom management, household support)
   - What social support have found helpful (e.g., health professionals, family and friends, other parents)
     - Have there been any online communities/social groups that you have found helpful? (e.g., Facebook parent groups)
   - Are there any types of online support/resources that you have found helpful for your physical health (e.g., sleep support, providing your own symptom management)
   - Have there been any online resources that you have found helpful for your mental health (e.g., coping strategies, managing stress and anxiety, spiritual support)
   - Have there been any practical support/services that you have accessed online that have helped you (e.g., information about transportation/housekeeping support services, financial support)
   - Have you come across any resources/information/strategies that have not helped/had negative consequences to your parenting your child through challenges?

6. **In a perfect world, regardless of what's available, what do you think would really make a difference in helping you support your child with CP?**

7. **Do you think that any of the resources that are available within either of the adult or youth Power over Pain portals would be helpful to address your needs as a parent of child with CP? If so, which ones and why?**
   Probes
   - Is there anything missing from that portal that would be helpful?
   - The adult PoP portal has webinars/workshops, which are really popular. Is this something that you would think would be helpful for you as a parent?
   - The PoP portals have a check in function, where youth can check-in and track their symptoms. Do you think a feature where parents could record and track their own symptoms would be helpful?
   - If there is a check-in feature for the parent, what would you like to be able to track?

8. **Is there anything else you would like to tell us about being a parent of a child with chronic pain or supporting the well-being of parents? Is there anything else that you think is essential to include/add to the PoP youth portal to support parents?**

9. **Do you have anything else to add that we haven't covered yet? Are there any specific topics you would like to discuss as we are wrapping up this interview?**
